# History of Brain Injury Alters Cerebral Haemodynamic Oscillations with Cardiac Influence

**DOI:** 10.3390/brainsci12111443

**Published:** 2022-10-26

**Authors:** J. Patrick Neary, Jyotpal Singh, Luke W. Sirant, Catherine A. Gaul, Steve Martin, Lynneth Stuart-Hill, Darren G. Candow, Cameron S. Mang, Gregory P. Kratzig

**Affiliations:** 1 Faculty of Kinesiology & Health Studies, University of Regina, Regina, SK S4S 0A2, Canada; 2 School of Exercise Science, Physical and Health Education, University of Victoria, Victoria, BC V8P 5C2, Canada; 3 Department of Psychology, University of Regina, Regina, SK S4S 0A2, Canada

**Keywords:** concussion, cerebral autoregulation, pre-frontal cortex oxygenation, blood pressure, wavelet transformation

## Abstract

(1) Background: Cerebral autoregulation is altered during acute mild traumatic brain injury, or concussion. However, it is unknown how a history of concussion can impact cerebral haemodynamic activity during a task that elicits an autoregulatory response. (2) Methods: We assessed cerebral haemodynamic activity in those with a history of three or more concussions. The study included 44 retired athletes with concussion history and 25 control participants. We recorded participants’ relative changes in right and left pre-frontal cortex oxygenation collected by near-infrared spectroscopy and continuous beat-to-beat blood pressure measured by finger photoplethysmography. Participants completed a 5-min seated rest followed by a 5-min repeated squat (10-s) stand (10-s) maneuver (0.05 Hz) to elicit a cerebral autoregulatory response. Wavelet transformation was applied to the collected signals, allowing separation into cardiac interval I (0.6 to 2 Hz), respiratory interval II (0.145 to 0.6 Hz), and smooth muscle cell interval III (0.052 to 0.145 Hz). (3) Results: Significant increases at cardiac interval I were found for the wavelet amplitude of oxy-haemoglobin and haemoglobin difference at the right pre-frontal cortex. No significant difference was found at the left pre-frontal cortex or the blood pressure wavelet amplitudes. (4) Conclusions: Contributions from cardiac activity to the pre-frontal cortex oxygenation are elevated when eliciting dynamic cerebral autoregulation in those with a history of three or more concussions.

## 1. Introduction

The majority of research in the concussion literature that has examined the effects of sport-related concussion (SRC) on cerebral physiology has focused on the acute injury phase. This research has documented significant negative effects on brain and cardiac physiology [1,2,3,4,5,6,7,8,9,10,11,12,13]. The acute physiological effects include altered cerebrovascular reactivity [10,14,15], cerebral oxygenation [16], baroreflex sensitivity [5,17], blood pressure variability [5,18], heart rate variability [3,19,20,21], dynamic cerebral autoregulation [22,23,24], and cardiac mechanics [25]. Furthermore, some research has shown that changes in functional connectivity are found in athletes with post-concussion syndrome [16,26,27,28], but not all research supports this contention [29]. It is possible that different analytical methodologies, protocols, ages, post-injury assessment time, and concussion history account for these discrepancies. Indeed, cardiorespiratory regulation and cerebral hemodynamics are influenced by many factors, including breathing pattern and being under anaesthesia [30].

Limited information is available on the long-term effects of sport-related concussion in retired contact sport athletes [23,31], and physiological studies of former athletes with a history of multiple concussions are particularly lacking. Some research has shown that contact sport athletes with a history of concussion can experience the same altered physiological mechanisms as those observed during the acute stage of concussion. For example, when assessing cerebral oxygenation using near infrared spectroscopy (NIRS), differences were documented between those with a history of sport-related concussion and active non-contact sport control participants [16,23,31]. Furthermore, NIRS has also been used successfully to assess cerebral autoregulation in adults [23,32,33,34,35]. Here, we applied NIRS to gain a better understanding of the potential mechanisms related to the recently reported long-term effects of multiple concussions on brain oxygenation and haemodynamics.

Given that biological signal oscillations vary in time, we elected to use wavelet transformation for our analyses [36]. Wavelet transformation is a time-frequency analysis methodology that provides optimal resolution for low and high frequencies and provides critical information about changes that occur in time without making prior assumptions of the data [37]. This makes wavelet analysis an ideal method to investigate biological systems in their natural resting state [38,39].

Based on the mounting research showing that the autonomic nervous system is affected in acute SRC, and that limited information is available on the long-term effects, the primary objective of this study was to examine changes in the cerebral autoregulatory response in retired contact sport athletes who had three or more concussions in their playing careers and compare them to retired non-contact sport athletes without a history of concussion. We used wavelet transformation analysis of the biological signals as this is a precise methodology to investigate biological oscillations over time [38,39]. We hypothesised that wavelet transformation of NIRS-based data could be used to discern physiological differences in the autoregulatory response in retired contact sport athletes with concussion when compared to control participants without concussion.

## 2. Materials and Methods

This study was conducted as part of a larger study to examine the long-term effects of sport-related concussion (SRC) on brain health in retired athletes [23]. Male contact sport participants (n = 84; 40–75 years) were recruited in Regina, SK, and Victoria, BC, Canada, with testing conducted at the Universities of Regina and Victoria. Ethics approval was acquired prior to conducting this study (REB#2017-032; REB#17-128). Prior to each participant signing the informed consent, the testing protocol and objectives were explained in detail.

Briefly, study participation involved one session in which participants completed a 5-min seated rest and 5-min repeated squat–stand protocol while continuous blood pressure and NIRS data were collected. From the total 84 participants who volunteered, 69 had clean blood pressure (BP) and cerebral haemodynamic oscillation waveforms from which the results are based. This sample included 44 participants with a history of sport-related concussion (SRC; at least 3 concussions in their playing careers; range = 3–20, median = 3 concussions) and 25 control participants (no concussions).

The SRC group included American football, soccer, rugby, and ice hockey players. CTR participants competed in water sports, running, tennis, soccer, cycling, and golf. To control for differences in fitness and athlete characteristics between groups, the inclusion criteria required that participants had played sports in their youth, and at the time of study participation, all participants still maintained an active lifestyle by engaging in physical activities a minimum of three times a week. Medical and concussion history, height (cm), and body mass (kg) were collected, including information on diet, caffeine, and alcohol consumption, exercise, sleep, and medications for the 24 h prior to testing. Both physician diagnosed and self-reported concussions were recorded for each participant. To ensure that participants were not experiencing concussion symptoms at the time of testing, the Sport Concussion Assessment Tool 5th edition (SCAT5) symptom scale [40] was completed. Participants did not consume any caffeine within 4 h, perform exercise within 6 h, or consume alcohol within 12 h of the study session, as these factors and time frames have all been shown to influence cerebrovascular metrics [41]. Volunteer participants experiencing any current symptoms or confirmed diagnosed concussion at the time of testing were excluded from this study.

The study session testing protocol has been described in detail elsewhere [23]. Briefly, the NIRS probes were placed 1 cm above each eyebrow, over the right and left pre-frontal cortex, on the lateral side of the supraorbital ridge to avoid the frontal sinus [4]. The probes were held in place by a dark coloured headband which also helped to avoid external infrared light from interfering with the optical (oxygenation) signals. The devices were connected via Bluetooth to the Oxysoft 3.0.97.1 software on laptop computers for data collection. For the majority of participants, two PortaLite devices (Artinis Medical Systems, Einsteinweg, The Netherlands) were used to monitor both the left and right pre-frontal cortices. Approximately 25% of the participants had their left pre-frontal cortex monitored using an OxyMon (Artinis Medical Systems, Einsteinweg, Netherlands) NIRS device which has identical functionality as the PortaLite device. These devices use continuous-wave near infrared light to assess relative changes in cerebral oxygenation parameters (micro-molar, µM) as well as the same data collection and analysis software. The NIRS probes use one receiver and 3 pairs of light emitting diodes (LED). The first pair of LEDs (760 and 843 nm) is located 30 mm from the receiver, the second pair (761 and 845 nm) is located 35 mm from the receiver, and the third pair (762 and 848 nm) is located 40 mm from the receiver. The cerebral oxygenation parameters included oxy-haemoglobin (HbO_2_), deoxy-Hb (HHb), total haemoglobin (tHb = HbO_2_ + HHb), and haemoglobin difference (HbDiff = HbO_2_ − HHb).

A Finapres NOVA (Finapres Medical Systems, Arnhem, The Netherlands) was used to measure continuous blood pressure (BP). Finger photoplethysmography BP was calibrated against brachial arterial pressure. The BP data was collected on a beat-by-beat basis. Of the 44 SRC participants, 42 participants had usable left and right pre-frontal cortex oxygenation oscillations measured using NIRS, and blood pressure waveform oscillations using finger photoplethysmography. A total of 25 CTR participants were included in the blood pressure assessment, and 18 had usable left and right NIRS oscillations.

In this study session, to assess differences in pre-frontal cortex oxygenation between the SRC and CTR groups, we used a squat–stand maneuver to elicit a dynamic cerebral autoregulatory (dCA) response [2,23,24,42]. Participants sat quietly for 5-min to establish resting baseline physiology. Prior to the squat–stand maneuver, the participants stood up for 1-min to allow the body to adjust to the standing position so that a new baseline flow-pressure equilibrium ‘set point’ could be established [43]. Thereafter, each participant completed the squat–stand maneuver at a squat rate of 0.05 Hz (cyclical 10-s squat followed by 10-s of standing), repeated 15 times for a total of 5 min [23,42]. During each squat–stand, participants were instructed to keep their head in a level plane by “looking straight forward” to avoid erroneous measurements related to a head tilt. A goniometer was used to ensure that the participant squatted to 90° at the knees. The participant was corrected if a squat was performed incorrectly and encouraged to follow the established protocol on subsequent squats.

The data were collected and amplified through PowerLab 16/32 and streamed into LabChart 7 Pro (AD Instruments, Colorado Springs, CO, USA). All signals before analysis were down sampled to 10 Hz, detrended using a moving average with a window size of 220 s and normalised by subtraction of their mean and divided by their standard deviation [23].

A wavelet transformation was used for our analyses as a major strength of using wavelet transformation is that it allows for the analysis of biological signals as they change with time due to physiological perturbations [44]. Specifically, the wavelet transformation can quantify and delineate the investigated interactions in both frequency and time domains [38,44]. As such, the wavelet transformation can convert the NIRS signals from time domain to a time-frequency domain [45,46], thus providing information on the main components of the time series in frequency domain by detecting spontaneous fluctuations, with the amplitude describing the activity intensity of the cortex at the brain regions [47]. Furthermore, when assessing NIRS signals, the use of wavelet can provide insights into cerebral autoregulation. Indeed, when undergoing a dynamic cerebral autoregulation eliciting task, such as repeated squat stands used in this study, it has been suggested that the quantified findings from wavelet transformation of NIRS provide valuable information about cerebral autoregulation that is not possible using other methods [48]. Thus, the separation into known physiological frequency intervals allows for an understanding of the mechanism associated with altered cerebral hemodynamic activity, given that the characteristic frequencies indicate possible regulatory mechanisms. Finally, when assessing NIRS and BP by wavelet, the original signal features are retained, which is attractive for data with time-varying features, as compared to other filtering methods [49]. We examined the cardiac interval (I: 0.6–2 Hz), respiratory interval (II: 0.145–0.6 Hz interval), and the smooth muscle cell interval (III: 0.052–0.145 Hz). Furthermore, the mother Morlet wavelet was used for its strong localisation of events in time and frequency due to its Gaussian shape [44]. The methodology for the wavelet transformation analyses used for this study has been described extensively, including what each frequency interval represents [38,39,44].

Briefly, the wavelet transformation is defined as follows:

$$W\left(s,t\right)=\frac{1}{\sqrt{s}}\;\int_{-\infty }^{+\infty }\varphi \;\left(\frac{u-t}{s}\right)g\left(u\right)du,$$

where *W* (*s*, *t*) is the wavelet coefficient, *g*(*u*) is the time series and *φ* is the Morlet mother wavelet, scaled by factors and translated in time by *t*. The Morlet mother wavelet is defined by the equation:

$$\varphi \left(u\right)=\frac{1}{\sqrt[{4}]{\pi }}\exp\left(-i2\pi u\right)\exp\left(-0.5u^{2}\right)$$

where *i* = √−1. This was followed by calculation of wavelet coefficients,

$$Χ\left(\omega_{k,\;}\;t_{n}\right)=X_{k,n}=a_{k,n}+ib_{k,n}\;$$

which represent instantaneous relative phase and absolute amplitude, defined as

$$\left|X_{k,n}\right|=\sqrt{a_{k,n}+b_{k,n}}\;$$

where *X_k,n_* of the wavelet transformation is a complex number (*a* and *b* are elements of this complex number); *k* = 1, 2, …, K and *n* = 1, 2, …, N, where K and N are natural numbers for the different number steps for frequency and time.

### Statistical Analysis

We used a conservative approach to our data analysis because normality tests such as the Shapiro–Wilk tend to reduce the statistical power when analysing smaller sample sizes. Thus, we used the Wilcoxon rank sum nonparametric test for all data [50] to avoid the assumption of normality.

## 3. Results

### 3.1. Pre-Frontal Cortex Oxygenation (NIRS Parameters)

**Figure 1:**
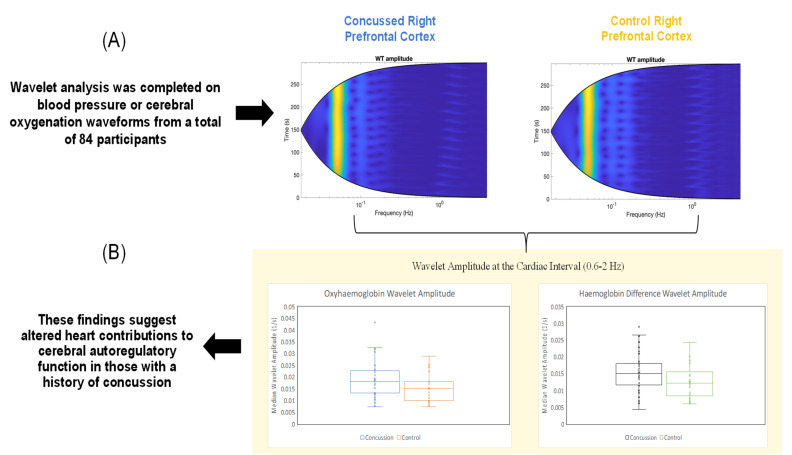
Central figure displays the wavelet transformation analysis. Panel (**A**) shows the frequency distribution (Hz) and amplitude changes in relation to time (seconds, s) for the right prefrontal cortex oxygenation for the concussed (SRC) and control (CTR) groups. Panel (**B**) shows the box−plots for the calculated values found in Table 1. The box−plots shown here represent the cardiac interval (0.6−2 Hz) amplitude which was significantly different between groups for both the oxy-haemoglobin and haemoglobin difference.

Table 1 represents the frequency response of the time-averaged wavelet transformation for the NIRS cerebral oxygenation and BP response. Significant between group differences in the relative changes in cerebral haemodynamics were found only at the right pre-frontal cortex. A significantly different increase (*p* = 0.04) in HbO_2_ was seen at interval 0.6 to 2 Hz (cardiac) in SRC group (median = 0.018, IQR = 0.009) compared to CTR group (median = 0.015, IQR = 0.008). Similarly, a significantly different increase (*p* = 0.03) in HbDiff was seen at interval 0.6 to 2 Hz (cardiac) in the SRC group (median = 0.015, IQR = 0.006) compared to CTR (median = 0.012, IQR = 0.006) (See Central Figure 1). No other significance between group differences were found at any intervals.

### 3.2. Blood Pressure (BP)

No significant differences were found in the wavelet transform BP amplitudes in any of the measured frequency intervals (cardiac, respiratory, smooth muscle cell).

## 4. Discussion

We hypothesised that the long-term effects of concussion in retired contact sport athletes could be detected from physiological measures. Our results support this contention; we found that dynamic cerebral autoregulation (dCA) reflected by pre-frontal cortex oxygenation was statistically different between adults with a history of multiple sport-related concussions and a healthy control group when assessed using wavelet transformation. Specifically, we provide new evidence that dCA is influenced by the cardiac contribution to maintaining cerebral autoregulation in those retired athletes with a history of concussion.

Using a repeated squat–stand maneuver that is documented to be an appropriate method to assess cerebral autoregulation [23,24,42], the current research results add to the emerging evidence of potential long-term consequences of multiple concussions in contact sport athletes. Previous research has also shown that neurovascular coupling [31], functional connectivity [16], and dCA [1,23] are altered long-term in those who experience multiple concussions throughout their sporting career. Together this research suggests that retired contact sport athletes who have experienced concussions must be cognisant of those head injuries and consider taking steps to mitigate the potential for additional head trauma.

Our results also add to the body of literature regarding the interaction between the heart and the brain (heart–brain Axis). There is a growing body of knowledge confirming that sport-related concussion has significant adverse effects on cardiac function in both acute and chronic post-concussion [25,51]. Thus, our observation that the cardiac interval I (0.6 to 2 Hz) was the frequency interval that significantly contributed to the relative changes in right pre-frontal cortex oxygenation in HbO_2_ and HbDiff in those with a history of concussion are notable (see Central Figure 1). The increased relative changes in pre-frontal cortex HbO_2_ and HbDiff of our concussion group in comparison to the controls reflects differences in the metabolic status and the brain’s response to the autoregulatory (squat-stand) challenge. Simply stated, the heart–brain axis is tightly coupled and our findings suggest that there is a long-term compensatory response from the heartbeat to cerebral haemodynamic activity after concussion. This hypothesis is supported by previous [51] and on-going research [25]. Furthermore, we speculate that our results shine light on the possible implications for cardiac health with ageing, and how concussions early in an athlete’s playing career may contribute to cardiac dysfunction later in life, i.e., ventricular ectopy [51].

Collectively, these results provide significant implications for the management of concussions early in life and later during playing careers in contact sports. For example, in the past there was less emphasis on the recovery period and when athletes could return-to-play safely post-concussion. The old adage was “suck-it up and get back out there”. The findings of this current study now highlight the importance of conducting baseline physiological testing at the beginning of a sporting season for any contact sport athletes. Recent research supports the use of baseline physiological testing for standardising pre-participation guidelines so that the medical staff responsible for getting players back-to-play safely, post-concussion, can provide an objective assessment of the players’ physiological status [5,41] to avoid early return-to-play and potential adverse complications.

## 5. Conclusions

Our data confirm the heart’s contributions to cerebral autoregulatory function, and suggests a physiological mechanism for the differences between retired sport contact athletes with a history of multiple concussions and those retired athletes without a history of concussion, i.e., the heart helps to maintain cerebral oxygenation when the body is stressed autonomically in those with a history of concussion. Future research integrating other physiological signals such cerebral blood flow velocity, blood pressure, cerebral oxygenation, ECG, pial artery, and subarachnoid space oscillations during dynamic autoregulatory and cerebrovascular reactivity-induced perturbations will add significantly to this line of research.

## Figures and Tables

**Table 1:** Time-averaged wavelet transformations for the right and left pre-frontal cortex cerebral haemodynamics and blood pressure responses.

		Right-Side Pre-Frontal Cortex			Left-Side Pre-Frontal Cortex			
	Interval	HbO_2_ (1/s)	tHb (1/s)	HbDiff (1/s)	HbO_2_ (1/s)	tHb (1/s)	HbDiff (1/s)	BP (1/s)
Injured	0.052–0.145	0.0976 (0.0308)	0.1016 (0.0294)	0.0959 (0.0385)	0.0939 (0.0290)	0.1002 (0.0372)	0.0975 (0.0265)	0.0613 (0.0150)
	0.145–0.6	0.0255 (0.0096)	0.0297 (0.0110)	0.0203 (0.0091)	0.0257 (0.0111)	0.0309 (0.0110)	0.0189 (0.0089)	0.0429 (0.0128)
	0.6–2.0	0.0180 (0.009) *	0.0200 (0.0098)	0.0150 (0.006) **	0.0159 (0.0081)	0.0178 (0.0117)	0.0140 (0.0063)	0.1050 (0.0320)
Control	0.052–0.145	0.0889 (0.0343)	0.0999 (0.0363)	0.0936 (0.0267)	0.0874 (0.0259)	0.0892 (0.0357)	0.0853 (0.0167)	0.0571 (0.0137)
	0.145–0.6	0.0291 (0.0170)	0.0363 (0.0206)	0.0198 (0.0146)	0.0229 (0.0207)	0.0295 (0.0253)	0.0170 (0.0150)	0.0423 (0.0174)
	0.6–2.0	0.0150 (0.008) *	0.0179 (0.0105)	0.0120 (0.006) **	0.0150 (0.0087)	0.0181 (0.0089)	0.0140 (0.0113)	0.1014 (0.0574)

Data are presented as median (interquartile range). HbO_2_: oxy-haemoglobin, tHb: total haemoglobin, HbDiff: haemoglobin difference, BP: Blood pressure. Frequency intervals are I: 0.6–2 Hz (cardiac interval), II: 0.145–0.6 Hz (respiratory interval), and III: 0.052–0.145 Hz (smooth muscle cell interval). * Significant (*p* < 0.05) between-group difference at interval 0.6–2 Hz for HbO_2_ right-side; ** Significant between-group difference at interval 0.6–2 Hz for HbDiff right-side.

## Data Availability

The data from this study cannot be accessed at this time.
